# Retrospective Analysis of Ugandan Men with Urethritis Reveals Mycoplasma genitalium and Associated Macrolide Resistance

**DOI:** 10.1128/spectrum.02304-21

**Published:** 2022-04-12

**Authors:** Johan H. Melendez, Justin Hardick, Annet Onzia, Tong Yu, Peter Kyambadde, Rosalind Parkes-Ratanshi, Edith Nakku-Joloba, Agnes Kiragga, Yukari C. Manabe, Matthew M. Hamill

**Affiliations:** a Division of Infectious Diseases, Johns Hopkins School of Medicine, Baltimore, Maryland, USA; b Infectious Diseases Institute, Makerere University College of Health Sciences, Kampala, Uganda; c Ministry of Health, National Sexually Transmitted Infections Control Program, Kampala, Uganda; National University Hospital

**Keywords:** *Mycoplasma genitalium*, NAAT, sexually transmitted infections, antimicrobial resistance

## Abstract

The rising rates of antimicrobial resistance (AMR) in Mycoplasma genitalium globally and the association of this sexually transmitted infection (STI) with cervicitis, urethritis, and HIV are potentially of great public health concern. Data on the epidemiology of M. genitalium in men in sub-Saharan Africa are limited. We sought to determine the prevalence of M. genitalium and macrolide resistance in men with urethritis in Kampala, Uganda. Self-collected penile-meatal swabs and/or urine samples from men with symptomatic urethritis (*n* = 250) were retrospectively analyzed for the presence of M. genitalium and macrolide resistance markers with the Aptima M. genitalium and *ResistancePlus* M. genitalium assays. Additionally, demographic and STI coinfection data were used to investigate associations with M. genitalium infection. M. genitalium was detected in 12.8% (32/250) of individuals; 40.6% (*n* = 13) had M. genitalium monoinfection. Mutations associated with macrolide resistance were detected in 10.7% (3/28) of participants. Coinfection with Neisseria gonorrhoeae was common (41.0%), but M. genitalium was more prevalent in participants without N. gonorrhoeae coinfection (*P* = 0.001). M. genitalium is common in Ugandan men with urethritis both as a monoinfection and as a coinfection with other curable STIs. Macrolide resistance was present and warrants further research on treatment outcomes and the association between untreated M. genitalium and subsequent morbidity.

**IMPORTANCE** Mycoplasma genitalium is a common sexually transmitted infection associated with urethritis in men. Little is known about M. genitalium infection in men with urethritis in Uganda. We report that 12% of participants in this study were positive for M. genitalium and that resistance to azithromycin, a macrolide antibiotic, is present. Furthermore, we show that either self-collected penile-meatal swabs or urine can be used for detection of M. genitalium.

## INTRODUCTION

Mycoplasma genitalium is a common, yet often underdiagnosed, sexually transmitted infection (STI) associated with nongonococcal urethritis (NGU) in men and poor sexual and reproductive health outcomes in women, including cervicitis, pelvic inflammatory disease (1), and increased risk of HIV acquisition (2, 3). The prevalence of M. genitalium in the general population in high-income countries is approximately 1.3% but higher (3.2%) in community-based men who have sex with men (MSM) and commercial sex workers (CSWs) (15.9%) in lower-income settings (4). In comparison to other STIs, there are limited data regarding M. genitalium infections from sub-Saharan Africa (SSA), especially in men. Previous studies from SSA including symptomatic men in South Africa, a circumcision trial in Kenya, symptomatic STI clinic attendees in Zimbabwe, and Nigerian MSM have reported urogenital M. genitalium prevalence of 17.3%, 9.9%, 3.5%, and 12.4%, respectively (5–8). In Uganda, studies regarding the prevalence of M. genitalium have been limited to women and female CSWs (3, 9–12). The only study involving Ugandan men was conducted as part of a randomized trial of male circumcision and found that male circumcision did not affect M. genitalium infection in female partners, but the study did not report on the prevalence of M. genitalium in men (13).

The increasing frequency (14–16) and specter (17) of macrolide resistance have decreased the value of macrolides as a first-line treatment for M. genitalium. Azithromycin is no longer recommended in first-line treatment of urethritis and cervicitis syndromes but remains one of the first-line regimens for the treatment of M. genitalium if macrolide sensitivity is determined using antimicrobial resistance (AMR) testing. A meta-analysis on the prevalence of mutations associated with M. genitalium macrolide resistance using data from 21 countries found an overall prevalence of 35.5%, increasing from less than 10% before 2010 to 51.4% in 2016 to 2017 (16). Macrolide resistance is mediated by mutations in the 23S rRNA gene (A2058 and A2059 [Escherichia coli numbering]), which prevent binding of the antibiotic to the 23S rRNA (1). The use of sequential treatment with doxycycline and azithromycin or moxifloxacin has been demonstrated to increase cure rates using a resistance-guided approach (18). It is recommended in several national guidelines including in Australia (19) and the United Kingdom (20). In 2021, the Centers for Disease Control and Prevention (CDC) recommended the use of sequential treatment (doxycycline and azithromycin) for macrolide-susceptible M. genitalium or doxycycline-moxifloxacin when macrolide resistance is confirmed or if macrolide resistance testing is not available (21). Data pertaining to AMR in M. genitalium from Africa are beginning to emerge but have been largely limited to South Africa, where low levels (0 to 1.1%) of azithromycin resistance have been reported (22–24). Considering the high levels of antimicrobial-resistant gonorrhea in Uganda (25, 26), understanding the epidemiology and AMR profile of M. genitalium infections is critical to help shape treatment guidelines, which have not been defined for M. genitalium infections in Uganda (27).

We retrospectively evaluated the prevalence of M. genitalium and macrolide resistance-associated mutations (MRMs) in urogenital samples collected from Ugandan men with urethritis in Kampala, Uganda. We performed a small comparison to evaluate the performance of the Aptima and the *ResistancePlus* assays for detection of M. genitalium utilizing male urine samples. Additionally, we sought to determine the association between M. genitalium and participants’ demographics, HIV status, and coinfection with other STIs.

## RESULTS

### Participants’ characteristics, sexual behaviors, and STI coinfections.

The characteristics of the 250 participants included in the study are shown in Table 1. The majority, regardless of M. genitalium positivity, were under the age of 24 (52.4%), were men who have sex with women (MSW) (95.2%), and were HIV negative (80%) and syphilis negative (90%). M. genitalium was detected in 12.8% (32/250) of participants by Aptima nucleic acid amplification test (NAAT). There were no significant differences in age, sexual orientation, and HIV and syphilis positivity by M. genitalium status. Compared to participants with a negative M. genitalium test, those with M. genitalium were significantly less likely to be coinfected with other curable STIs (*P* = 0.008).

**TABLE 1:** Demographic characteristics of study participants, STIs, and association with M. genitalium^*a*^

Characteristic	M. genitalium positive (N = 32), *n* (%)	M. genitalium negative (N = 218), *n* (%)	*P* value overall
Age, median, yr [IQR]	24.0 [21.0; 32.2]	24.0 [22.0; 32.0]	0.619

Age group (yr)			0.951
16–20	4 (12.5)	30 (13.8)	
21–24	13 (40.6)	84 (38.5)	
25–34	10 (31.2)	60 (27.5)	
≥35	5 (15.6)	44 (20.2)	

Sexual orientation			0.187
MSW	29 (90.6)	209 (95.9)	
MSM/MSMW	3 (9.4)	9 (4.1)	

HIV			0.962
Negative	25 (78.1)	175 (80.3)	
Positive	7 (21.9)	43 (19.7)	

Syphilis			0.338
Negative	27 (84.4)	198 (90.8)	
Positive	5 (15.6)	20 (9.2)	

Neisseria gonorrhoeae	13 (40.6)	154 (70.6)	**0.001** ^*b*^
Chlamydia trachomatis	8 (25.0)	49 (22.6)	0.937^*b*^
Trichomonas vaginalis	2 (6.2)	4 (1.8)	0.172

UDS-associated coinfections			**0.008**
At least one other STI	19 (59.4)	178 (81.7)	
No other STIs	13 (40.6)	40 (18.3)	

a N, number; IQR, interquartile range; UDS, urethral discharge syndrome; MSW, men who have sex with women; MSM, men who have sex with men; MSMW, men who have sex with men and women.

b The number of samples (N. gonorrhoeae [*n* = 249], C. trachomatis [*n* = 249]) included in the analysis was less than 250 because of invalid Aptima results. M. genitalium prevalence is based on Aptima results. M. genitalium was detected in 93.8% (30/32) of paired penile-meatal swabs and urine; two participants had discordant results (urine positive but negative in the swab sample and vice versa). Bold indicates statistical significance.

Table 2 describes the age, sexual behavior, STI coinfections, 23S rRNA MRMs, antimicrobial treatments, and symptom resolution outcomes for participants with M. genitalium. In 40.6% (*n* = 13) of M. genitalium-positive participants, M. genitalium was the only curable STI detected by the Aptima NAAT. However, the majority (59.4% [19/32]) of participants with M. genitalium were coinfected with at least one additional curable STI. Coinfections of M. genitalium with Neisseria gonorrhoeae, Chlamydia trachomatis, or Trichomonas vaginalis were found in 28.1% (*n* = 9/32), 12.5% (*n* = 4/32), and 6.3% (*n* = 2/32), respectively. M. genitalium, C. trachomatis, and N. gonorrhoeae coinfection was found in 12.5% (*n* = 4/32).

**TABLE 2:** Demographics, STIs, treatment, and outcome of M. genitalium-positive participants^*a*^

PID	Age (yr)	Sexual orientation	Urethritis-associated STI(s)	AZM resistance 23S rRNA	Empiric treatment received^*b*^	Resolution of symptoms
			1 STI			
MN-021	24	MSM	M. genitalium	Mutant	1, 2, 4	F-U-3
MN-023	25	MSMW	M. genitalium	Mutant	1, 2, 5	F-U-2
MN-034	19	MSW	M. genitalium	Wild type	1, 2, 5	F-U-1
MN-156	38	MSW	M. genitalium	Wild type	6, 2	F-U-1
MN-198	21	MSW	M. genitalium	Wild type	1, 2, 5	F-U-1
MN-207	27	MSW	M. genitalium	Wild type	1, 2, 5	F-U-1
MN-227	42	MSW	M. genitalium	Wild type	1, 2, 5	F-U-2
MN-197	21	MSW	M. genitalium	Wild type	1, 2, 5	F-U-2
MN-236	24	MSW	M. genitalium	Wild type	1, 2, 5	F-U-2
MN-120	22	MSW	M. genitalium	Wild type	1, 2, 5	F-U-3
MN-248	27	MSW	M. genitalium	Wild type	1, 2, 5	F-U-3
MN-086	28	MSW	M. genitalium	Wild type	3, 2, 7	NR-F-U-3
MN-039	34	MSW	M. genitalium	No data	1, 2, 5	F-U-2
			2 STIs			
MN-018	33	MSW	M. genitalium and C. trachomatis	Wild type	1, 2, 5	F-U-1
MN-077	21	MSW	M. genitalium and C. trachomatis	Wild type	1, 2, 5	F-U-3
MN-121	17	MSW	M. genitalium and C. trachomatis	Wild type	1, 2, 5	LTFU
MN-069	22	MSW	M. genitalium and C. trachomatis	Wild type	1, 2	NR-F-U-3
MN-027	32	MSW	M. genitalium and N. gonorrhoeae	Wild type	1, 2, 5	F-U-1
MN-049	22	MSW	M. genitalium and N. gonorrhoeae	Wild type	1, 2	F-U-1
MN-051	28	MSW	M. genitalium and N. gonorrhoeae	Wild type	1, 2	F-U-1
MN-052	36	MSW	M. genitalium and N. gonorrhoeae	Wild type	1, 2, 4	F-U-1
MN-167	34	MSW	M. genitalium and N. gonorrhoeae	Wild type	3, 2	F-U-1
MN-231	38	MSW	M. genitalium and N. gonorrhoeae	Wild type	1, 2, 5	F-U-3
MN-043	21	MSW	M. genitalium and N. gonorrhoeae	Wild type	1, 2	F-U-3*
MN-030	20	MSM	M. genitalium and N. gonorrhoeae	No data	1, 2, 5	F-U-1
MN-153	24	MSW	M. genitalium and N. gonorrhoeae	No data	1, 2, 5	F-U-1
MN-188	23	MSW	M. genitalium and T. vaginalis	Wild type	1, 2, 5	NR-F-U-3
MN-229	40	MSW	M. genitalium and T. vaginalis	No data	1, 2, 5	F-U-2
			3 STIs			
MN-058	20	MSW	M. genitalium, C. trachomatis, and N. gonorrhoeae	Mutant	1, 2, 5	F-U-1
MN-063	23	MSW	M. genitalium, C. trachomatis, and N. gonorrhoeae	Wild type	1, 2, 5	F-U-1
MN-139	21	MSW	M. genitalium, C. trachomatis, and N. gonorrhoeae	Wild type	1, 2, 5	F-U-1
MN-050	29	MSW	M. genitalium, C. trachomatis, and N. gonorrhoeae	Wild type	6, 2	LTFU

a PID, patient identifier; MSW, men who have sex with women; MSMW, men who have sex with men and women; MSM, men who have sex with men; AZM, azithromycin; No data: The 23S rRNA genotype could not be determined by either the *ResistancePlus* assay or Sanger sequencing. F-U-1, follow-up 1 (7 days posttreatment); F-U-2, follow-up 2 (14 days posttreatment); F-U-3, follow-up 3 (21 days posttreatment); NR-F-U-3, symptoms not resolved as of follow-up visit 3; LTFU, lost to follow-up; *, no follow-up 2 visit.

b Treatment key: 1, cefixime; 2, doxycycline; 3, ceftriaxone; 4, azithromycin; 5, metronidazole; 6, other (cefuroxime, cefotaxime); 7, gentamicin.

### Comparison of urine Aptima and *ResistancePlus* results.

There was a 90.6% (29/32) agreement between the Aptima assay and the *ResistancePlus* assay for detection of M. genitalium. The urine samples of 28 participants tested positive by both assays, and one urine sample tested negative by both assays despite the corresponding penile swab testing positive by Aptima assay. Three urine samples tested positive for M. genitalium with the Aptima assay but M. genitalium negative with the *ResistancePlus* assay.

### Detection of 23S rRNA MRMs using the *ResistancePlus* assay.

Of the 32 urine samples from Aptima M. genitalium-positive participants, 87.5% (*n* = 28) had sufficient DNA and provided conclusive 23S rRNA genotyping results. The presence of 23S rRNA MRMs was confirmed in 10.7% (3/28) of samples by Sanger sequencing; of these, 66% (2/3) were from participants who self-identified as MSM (Table 2). Sanger sequencing revealed that two samples harbored the 23S rRNA A2059G mutation and one sample had the A2058G mutation. Two of these samples were classified as 23S rRNA mutants by the *ResistancePlus* M. genitalium assay, and the third sample was indeterminate. The *ResistancePlus* M. genitalium assay identified wild-type 23S rRNA sequences, suggestive of macrolide susceptibility, in 25 of the samples from M. genitalium-positive participants. The macrolide susceptibility profile (23S rRNA genotype) of four M. genitalium-positive participants could not be determined by either the *ResistancePlus* M. genitalium assay or Sanger sequencing.

### Antimicrobial treatment and resolution of UDS symptoms.

Table 2 describes the antimicrobial treatment and self-reported urethral discharge syndrome (UDS) symptom resolution for M. genitalium-positive participants. The majority (87.5% [*n* = 28]) of participants with M. genitalium were empirically treated for UDS on the day of their clinic visit with a single dose of cefixime plus doxycycline (7 days) as recommended by the 2016 Ugandan STI treatment guidelines (27). Azithromycin was empirically prescribed to 6.3% (2/32) of those who subsequently tested positive for M. genitalium. The majority (86.7% [26/30]) of participants with M. genitalium, including 92.3% (12/13) of participants with M. genitalium monoinfection, reported resolution of UDS symptoms within 21 days of treatment; three participants reported symptoms during the last follow-up phone call visit (21 days posttreatment). All three participants with macrolide-resistant (mutant 23S rRNA) M. genitalium reported resolution of symptoms by the third (day 21) follow-up phone call or earlier; in two of these participants, M. genitalium was the only STI detected by Aptima NAAT.

## DISCUSSION

The data presented here describe the prevalence of M. genitalium in men with UDS, which is similar to that found in traditional high-risk groups, CSWs and MSM (4). Among participants who tested positive for M. genitalium, 40% were monoinfected. Taken together, these findings suggest that M. genitalium is an important pathogen in the etiology of male UDS (28).

In our study of men attending community health centers, gonorrhea was the most prevalent STI; in those infected with N. gonorrhoeae, the overall prevalence of M. genitalium was 7.8%, which is similar to the prevalence (7.6%) reported in men attending a sexual health clinic in Sydney, Australia (29). Although, N. gonorrhoeae infection was very common, we found that N. gonorrhoeae was more prevalent in men who were not infected with M. genitalium (70.6%) than in men who were coinfected with M. genitalium (40.6%). This difference may reflect differential exposure to sexual networks with different N. gonorrhoeae prevalence, behavioral factors such as frequency of condom use, prior antimicrobial exposure, or as-yet-unidentified M. genitalium-associated inhibition of N. gonorrhoeae. Larger studies are required to explore this association. Despite a 20% HIV positivity rate in our study, we did not find an association between HIV infection and M. genitalium as has been previously reported in other African studies (30, 31). The small number of HIV- and M. genitalium-positive participants in our study limits the exploration of associations. However, the association of M. genitalium and HIV acquisition in Ugandan women (3, 9) implies that case-control or prospective studies with a larger sample of participants with M. genitalium are warranted to establish relationships with potential risk factors, such as living with HIV.

The reported prevalence of macrolide-resistant M. genitalium strains has increased in many parts of the world over the last decade (16), but recent studies from Africa have reported various levels of resistance ranging from no resistance (22, 23) to <1.5% (24). Our study identified 10.7% of samples with macrolide resistance, which is comparable to that seen in high-risk women in Kenya (32). In comparison to the prevalence of macrolide resistance in other countries, which can be as high as 80% (33–35), our data suggest that macrolide resistance is emerging in Uganda, which is not surprising considering the lack of antimicrobial stewardship and high rates of antimicrobial-resistant gonorrhea (25). Although the number of macrolide-resistant M. genitalium infections in our study was small (*n* = 3), they were more common in MSM than in MSW, which is consistent with previous reports (36, 37).

Globally, management and control of M. genitalium are challenging for a variety of reasons, including the role of asymptomatic infection in disease processes, undertesting, and AMR. In Uganda, where M. genitalium is not addressed in current STI management guidelines, treatment of M. genitalium with doxycycline-based UDS treatment is inadequate. The high proportion of individuals with multiple UDS-associated STIs might confound posttreatment symptom resolution of those M. genitalium infections. In our study, however, the majority of participants with M. genitalium, including those with other STI coinfections, reported resolution of symptoms after receiving treatment for UDS in accordance with the Ugandan treatment guidelines. All of the participants with an M. genitalium monoinfection received doxycycline treatment, which could have helped to alleviate symptoms by reducing bacterial burden (38). The longer-term durability of clinical cure and whether the M. genitalium infection was eradicated are not known. A controlled clinical trial showed that only 33% of M. genitalium-positive patients eradicated the M. genitalium infection following treatment with doxycycline (39). Therefore, the high rate of clinical cure, based on symptom resolution, in our study is at odds with previous studies on the effectiveness of doxycycline for the treatment of M. genitalium.

Our study found excellent concordance between self-collected penile-meatal swabs and urine for the detection of M. genitalium. Laboratory diagnostics are not used in the syndromic case management of UDS in Uganda; however, the ability to collect either urine or self-collected penile swabs could expand sample choices for the implementation of future surveillance programs for M. genitalium and AMR, similar to the N. gonorrhoeae surveillance programs.

The limitations of our study include the lack of asymptomatic participants to allow for characterization of symptomatic and asymptomatic M. genitalium infections. Our study included a small number of M. genitalium-positive samples. Larger studies are warranted to better define the epidemiology of M. genitalium and AMR in Uganda. Data on resolution of symptoms were based on self-report, and test-of-cure sampling was not performed on any of the participants. Patients with UDS attending community clinics in Kampala are treated empirically, and reliable M. genitalium coverage is not included in UDS treatment. We were unable to perform a comparison between self-collected and clinician-collected penile swabs for detection of M. genitalium. However, urine has been shown to be a suitable specimen type for detection of STIs, especially in symptomatic men. Testing with the *ResistancePlus* M. genitalium assay was performed using a modified extraction protocol, which could have affected assay sensitivity. However, over 90% of Aptima M. genitalium-positive samples also tested positive with the *ResistancePlus* assay, suggesting that modifications to the extraction protocol did not significantly affect assay performance. Lastly, all of the samples were collected in Kampala, Uganda, albeit from 6 different clinics, from primarily self-reported MSW, thus limiting the generalizability of these results.

In conclusion, the results of this study add to our knowledge of M. genitalium in Africa by providing baseline data on the prevalence of M. genitalium and macrolide resistance in symptomatic men with urethritis in Kampala, Uganda. M. genitalium was prevalent as monoinfection and as a coinfection. Macrolide resistance may be more common in MSM than MSW. Self-collected penile-meatal swabs are a suitable specimen type for M. genitalium testing and AMR analysis and may be useful in research studies and surveillance programs where facilities for collection and transportation of urine are limited. M. genitalium, which appears to be an important causative agent of UDS, is probably not currently adequately covered by syndromic management guidelines in Uganda. This observation is at odds with the clinical cure, based on self-report symptom resolution, observed in our study even in those with 23S rRNA MRMs; this observation requires further exploration in prospective studies. Additional studies are warranted to better define the epidemiology of M. genitalium and the relationship between community macrolide use and AMR and longitudinal associations between incident M. genitalium, AMR, and subsequent morbidity.

## MATERIALS AND METHODS

Participants were recruited, through the Enhanced Gonococcal Antimicrobial Surveillance Program (EGASP), at six government health centers in Kampala, Uganda, to participate in a study evaluating the analytical performance of a new diagnostic platform for gonorrhea (40). Men with urethritis were recruited between October 2019 and November 2020 (recruitment was suspended between March and July 2020 due to COVID lockdown), their consent was obtained, and they were asked to self-collect penile-meatal swabs and urine. Blood was collected by a research nurse as well as demographic and behavioral data, which were collected via questionnaire. Prior to study enrollment, participants were treated or prescribed treatment empirically by clinic staff in accordance with Ugandan treatment guidelines (27), which indicate dual treatment with oral cefixime and doxycycline for UDS. Follow-up phone calls were made to participants on days 7, 14, and 21 after the enrollment visit.

### Laboratory testing in Kampala.

HIV testing was performed using sequential point-of-care (POC) HIV antibody tests, including the Determine (Alere, Waltham, MA), and Stat-Pak (Chembio, NY) assays, with SD-Bioline (Standard Diagnostics, Giheung-gu, Republic of Korea) as a tie breaker. Syphilis testing was performed using the Laborex treponemal antibody test (Zheijing Orient Gene Biotech Co. Ltd., China). All tests were performed according to manufacturer’s instructions.

### Detection of M. genitalium by Aptima nucleic acid amplification test.

The self-collected penile-meatal swabs were eluted in phosphate-buffered saline (PBS), frozen, and together with paired, frozen, urine samples sent to the Johns Hopkins University for additional testing. Two hundred microliters of the swab elution was placed in the multitest swab transport medium (STM). Urine samples (≈2 mL) were transferred to urine specimen transport tubes per manufacturer’s instructions. Swab and urine samples were tested for M. genitalium as well as Neisseria gonorrhoeae, Chlamydia trachomatis, and Trichomonas vaginalis using the Aptima (Hologic, San Diego, CA, USA) M. genitalium transcription-mediated amplification-based Research Use Only, Combo 2 C. trachomatis/N. gonorrhoeae, and T. vaginalis assays, respectively. Samples with invalid Aptima C. trachomatis, N. gonorrhoeae, T. vaginalis, or M. genitalium results were retested; samples with repeated (tested twice) invalid Aptima results were excluded from the analysis.

### *ResistancePlus* testing for M. genitalium and macrolide resistance.

For *ResistancePlus* testing, urine samples were selected for the analysis because (i) the majority of previous studies have used urine, (ii) sample availability was high, and (iii) utilizing urine allowed for repeat testing, if necessary. Urine samples from participants who tested positive for M. genitalium with the Aptima NAAT underwent testing with the *ResistancePlus* M. genitalium assay (SpeeDx, Sydney, Australia) to assess concordance with the Aptima M. genitalium assay and to determine if any of the M. genitalium-positive samples contained 23S rRNA MRMs. Due to supply limitations caused by the COVID-19 pandemic, the DNA extraction step for *ResistancePlus* M. genitalium testing was modified as described below and DNA was extracted utilizing the MagNA Pure LC V 1.0 instrument (Roche Diagnostics, Indianapolis, IN). In accordance with manufacturer instructions, *ResistancePlus* amplification reactions were performed on the ABI 7500 Fast DX real-time PCR instrument (Applied Biosystems, Foster City, CA), and sample validity and assay results were determined through FastFinder RUO v3.5.8 (UgenTec, Hasselt, Belgium) analysis.

Using a modified DNA extraction protocol, we performed three sample analyses with the *ResistancePlus* M. genitalium assay, a primary analysis and two subset analyses. The primary analysis utilized 200 μL of sample (193 μL of urine + 7 μL of internal control [IC] [SpeeDx, Sydney, Australia]) from Aptima M. genitalium-positive participants. The second set of analyses involved a large-volume (980 μL urine + 20 μL of IC) analysis of a subset of samples (*n* = 12) that were performed to determine if an increase in sample volume would resolve *ResistancePlus* M. genitalium-negative, Aptima M. genitalium-positive results (*n* = 8) and to confirm detection of 23S rRNA mutations (*n* = 2). Two additional samples were used as controls in the second set of analyses to ensure assay reproducibility when the sample volume increased from 200 μL to 1,000 μL. The third analysis was a further attempt to resolve any remaining *ResistancePlus* M. genitalium-negative, Aptima M. genitalium-positive results (*n* = 2) by analyzing 1,000 μL of sample (980 μL of urine in Aptima transport buffer + 20 μL of IC) that produced the initial Aptima M. genitalium-positive result. Three samples were also included as controls to ensure *ResistancePlus* M. genitalium result reproducibility when switching the sample matrix from urine to Aptima transport buffer.

### 23S rRNA Sanger sequencing.

The *ResistancePlus* M. genitalium assay has been shown to have excellent sensitivity and specificity for detection of 23S rRNA MRMs (41); therefore, Sanger sequencing was performed only on a subset of 25% (8/32) of Aptima M. genitalium-positive urine samples. Samples were chosen as wild-type controls (*n* = 3); to identify the 23S rRNA MRMs detected by the *ResistancePlus* assay (*n* = 2); or to resolve persistent *ResistancePlus*-negative, Aptima M. genitalium-positive results (*n* = 3). 23S rRNA PCR was performed in 30-μL reaction mixtures as previously described (42) with the following modifications: 15 μL of PowerUp SYBR green master mix (Applied Biosystems, Foster City, CA), 2 μL of each 10 μM primer, 1 μL of distilled water (dH_2_O), and 10 μL of sample extract. Amplifications were performed on the ABI Quant Studio 12K Flex using the following cycling conditions: 95°C for 20 s, 40 cycles of 95°C for 1 s and 60°C for 20 s, and melt curve analysis to confirm the presence of PCR product. Sanger sequencing was performed by the Johns Hopkins University School of Medicine Genetic Resources and Core Facility (GRCF) (RRID SCR-018669) in accordance with GRCF protocols. Samples that did not produce sequencing results, or were of poor sequencing quality, were resequenced using the same amplification and sequencing protocol with an alternate set of sequencing primers, which produced a longer PCR amplicon (785 bp): 23S-785F 5′AGTGAACGAGTGATCAAGTAGC3′ and 23S-785R 5′TCTAAATACGATTTCCAACCG3′.

### Ethical approval.

This study was approved by the Johns Hopkins University School of Medicine Institutional Review Board (IRB approval no. 00215298) and in Uganda by the Joint Clinical Research Center (protocol reference number JC0919) and the Ugandan National Council for Science and Technology (study number HS455ES). Written informed consent was obtained from all participants prior to the commencement of study procedures and data collection.

### Data analysis.

Continuous ages by M. genitalium positivity were compared using the Kruskal-Wallis test. Differences in other categorical variables (age group, sexual orientation, infection with HIV, syphilis, and coinfection with other STIs) by M. genitalium result were compared using the chi-square test if frequencies in all cells were greater than 5 and Fisher’s exact test if frequency in any cell was less than 5. Differences were considered statistically significant at 0.05. Analyses were conducted in R (43).
